# EMOTIONAL ADJUSTMENTS OF CUSTODIAL GRANDPARENTS LIVING WITH COVID-19: A NATIONAL PERSPECTIVE

**DOI:** 10.1093/geroni/igad104.1740

**Published:** 2023-12-21

**Authors:** Deborah Whitley, Susan Kelley, Christine Fruhauf, Andrea Smith, Bert Hayslip

**Affiliations:** Georgia State University, Atlanta, Georgia, United States; Georgia State University, Atlanta, Georgia, United States; Colorado State University, Fort Collins, Colorado, United States; Western Michigan University, Ada, Michigan, United States; University of North Texas, Denton, Texas, United States

## Abstract

Living with COVID-19 demands has created emotional challenges for grandparents raising grandchildren. The aim of this study was to explore the emotional adjustment of custodial grandparents’ caregiving under COVID demands (e.g., masking, hand washing, social distancing) (N=145; age range 30-79 years, Mage = 61.4 years, SD = 8.5). A national sample of grandparents were surveyed using a multi-scale index of psychological distress, reliance on intrapersonal resources during the pandemic, as well as sociodemographics. An initial hierarchical regression analysis of caregivers’ emotional adjustment was conducted, wherein (1) sociodemographic variables were first entered as a set followed by 2) a 17-item measure of COVID demands (alpha = .73), 3) two indices of grandparent intrapersonal resources [resilience and empowerment/social support and religious coping], and 4) two indices of psychosocial stress [parental distress, health, social/emotional and loneliness/parental distress, parent-child dysfunction, difficult child and parenting challenges]. The overall model was statistically significant (F 11, 123 = 22.98, p < .01), accounting for 64% of the variance in grandparent adjustment difficulties. While sociodemographics and COVID demands failed to predict emotional distress, both categories of grandparent intrapersonal resources (p < .01), and both categories of psychosocial stress (p < .01) did so. Subsequent correlational findings indicated that intrapersonal resources (social support and religious coping) mediated the relationship between COVID demands and emotional adjustment. These findings suggest that while grandparent caregivers experienced significant emotional distress during the pandemic, reliance upon the support of others as well as their own intrapersonal resources may be instrumental in managing such distress.

